# Preventing Acute Respiratory Distress Syndrome (ARDS) and to avoiding intubation in patients with COVID-19: an experience from a reanimation service in Morocco

**DOI:** 10.11604/pamj.supp.2020.37.1.22747

**Published:** 2020-09-10

**Authors:** Jaber El Kaissi, Noureddine Jebbar, Abdellatif Diai, Ali Zinebi, Jaouad Laoutid

**Affiliations:** 1 Anesthesiology & Intensive care Department, Moulay Ismail Military Hospital, Meknes, Morocco,; 2 Faculty of Medicine, Sidi Mohammed Ben Abdellah University, Fes, Morocco,; 3 Internal medicine Department, Moulay Ismail Military Hospital, Meknes, Morocco

**Keywords:** COVID-19, awake, prone position

## To the editors of the Pan African Medical Journal

The emergence of the novel coronavirus has been declared first in Wuhan, China in December 2019, and rapidly spread around the world. Then, it was declared as pandemic by the World Health Organization in March 12^th^, 2020 [1,2]. COVID-19 is defined by WHO as the respiratory disease caused by the newly emergent virus. In severe forms of illness COVID-19 can be complicated by Acute Respiratory Distress Syndrome (ARDS). 14 % of patients infected are likely to develop severe illness, and 5 % will require admission in intensive care units [3]. In fact, between the period of March 1^st^, and March 11^th^, 2020, between 9 % and 11 % of actively infected patients in Italy needed intensive care units [4]. If pandemic spreading continues at this speed, many countries, even those with the most efficient health care systems will be unable to face the massive need in intensive care beds, especially with the lack of medical equipment and medical personnel. It is known that ARDS in daily practice leads to an additional workload, which may become an obstacle to provide the right healthcare during the epidemic context with the massive admissions in ICU. Hence, avoiding the development of ARDS in Coronavirus disease would be a compelling idea to decrease saturation of intensive care units, but would that be possible?

We have read with interest the paper of Qin sun *et al*. in which they advise the health professionals to recognize cases of high-risk patients early. They have screened patients by monitoring daily heart rate, SpO_2_, and respiratory rate and looking for any sign of organ failure. And once a patient is considered high risk (SpO_2_93% under room air, RR> 30/min, HR > 120/min), rapid and robust intervention is engaged to avoid intubation. And among the proposed methods, there was the awake prone position [5]. Concerning the situation in our country, the epidemic is still in the beginning, and to face it, our hospital has set up two departments managed by two teams, one for mild illness and the other for severe cases and cases requiring critical care. We have asked to 3 patients hospitalized in mild illness side and who have presented desaturation under 93% and Respiratory rate up to 30 to have prone position. The first one was a 75 years old man with chronic obstructive pulmonary disease. The second one is a diabetic 70 years old woman, and the third case is a diabetic 66 years old woman. We have noticed that in all patients, only one man didn´t respect the instructions, and, as a result, he has developed ARDS, transferred to ICU and got intubated. For the other patients, increasing of saturation under room air has been noticed, with a progressive reduction in needs for oxygen and amelioration of the clinical condition of patients.

**Figure 1:**
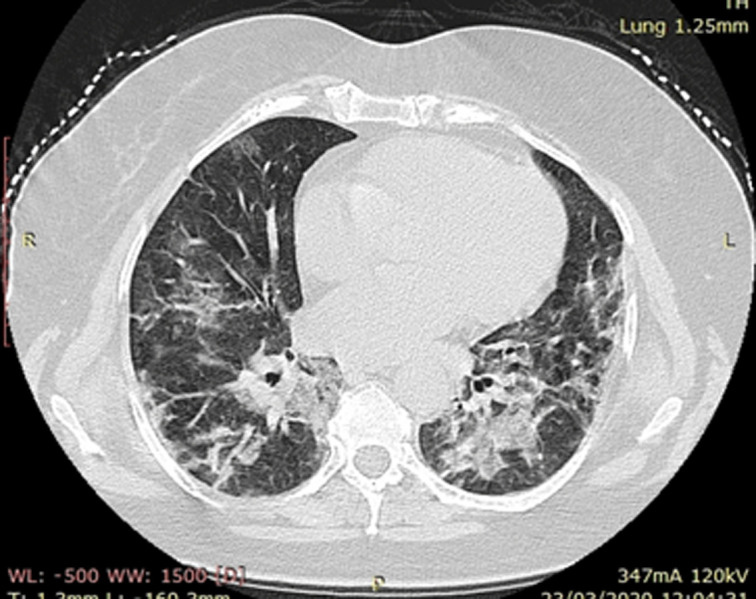
admission chest computed tomography

**Figure 2:**
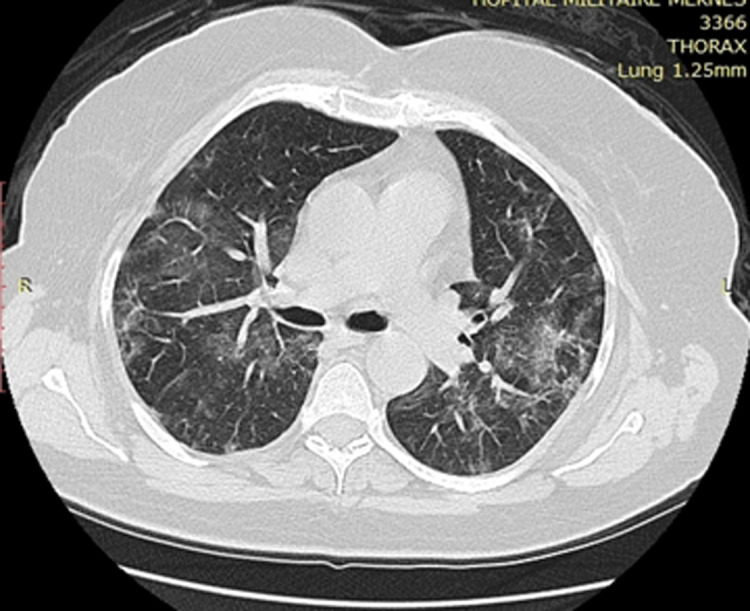
control chest computed tomography

The effect of the prone position was more marked and encouraging in the third patient. Chest computed tomography in admission has shown extension of bilateral ground-glass opacities to more than 60% from the total lung surface area (Figure 1). And after a week of spontaneously breathing prone position, control computed tomography has shown a definite improvement, less than 25% of bilateral ground-glass opacities (Figure 2). Before hearing about the technique, we had already admitted a young 47 years female with no medical background. This one had similar admission computed tomography findings that the third patient, and she wasn´t asked to have prone position. Her condition had deteriorated and she got intubated and developed ARDS. We have predicted the same thing for the third patient, but prone position has changed the outcome. Prone position has demonstrated significant benefit by reducing mortality in ventilated patients by improving ventilation-perfusion matching, increasing end-expiratory volume, and improving oxygenation [6]. The aim of using this technique in spontaneously breathing patients is to invest those properties in improving outcomes in patients with a severe case of coronavirus disease.

Valter has examined awake prone position in 4 patients in a case report published. The effects of pronation on arterial blood gas and clinical condition were registered. He found a rapid increase in pa02, and intubation was avoided in all patients [7]. Scaravilli *et al*. have evaluated in a retrospective study the effects of prone position on 15 non-intubated patients. They found that pronation was associated with significant improvement in oxygenation. They have also realized blood gas before, while pronation, and after. PaO_2_ was higher during pronation, and saturation was increasing in all patients. Paco_2_ and pH were not affected by pronation, and hemodynamic parameters were not changed [8]. The study of Scaravilli concerned patients immunocompromised in most of them, especially those who are at risk of developing severe coronavirus disease if they got infected. Scaravilli has shown good feasibility and good tolerance of the awake prone position, in 49 pronation, only two were interrupted due to patient intolerance. The procedure could be weaning for patients, and it may lead to intolerance. The medical staff should explain well the process to patients for better therapeutic observance. Moreover, there are no clear recommendations about the time duration of pronation or the use or not of supportive cushions. In our case, patients have been laid directly to bed, and there was good tolerance for all patients, concerning the duration, patients were asked to get the position the maximum of time they can. Awake prone position can be a good alternative, especially when we know that non-invasive ventilation is not recommended in COVID-19 patients because of aerosol-generating risk of this procedure that may expose healthcare to a higher infection danger [9]. Reports over the application of prone position in spontaneously breathing patients are still limited; it seems that this technique may help relieve congestion in ICU. We need more trials in this direction and we invite by the occasion teams in charge of COVID-19 units to consider this technique to prevent intubation and mechanical ventilation.
